# An influenza reassortant with polymerase of pH1N1 and NS gene of H3N2 influenza A virus is attenuated *in vivo*

**DOI:** 10.1099/vir.0.039701-0

**Published:** 2012-05

**Authors:** Holly Shelton, Matt Smith, Lorian Hartgroves, Peter Stilwell, Kim Roberts, Ben Johnson, Wendy Barclay

**Affiliations:** 1 Division of Infectious Diseases, Imperial College London, St Mary’s Campus, London, UK; 2 Health Protection Agency Colindale, 61 Colindale Avenue, London, UK

## Abstract

Influenza viruses readily mutate by accumulating point mutations and also by reassortment in which they acquire whole gene segments from another virus in a co-infected host. The NS1 gene is a major virulence factor of influenza A virus. The effects of changes in NS1 sequence depend on the influenza polymerase constellation. Here, we investigated the consequences of a virus with the polymerase of pandemic H1N1 2009 acquiring an NS gene segment derived from a seasonal influenza A H3N2 virus, a combination that might arise during natural reassortment of viruses that currently circulate in humans. We generated recombinant influenza viruses with surface HA and NA genes and matrix M gene segment from A/PR/8/34 virus, but different combinations of polymerase and NS genes. Thus, any changes in phenotype were not due to differences in receptor use, entry, uncoating or virus release. In Madin–Darby canine kidney (MDCK) cells, the virus with the NS gene from the H3N2 parent showed enhanced replication, probably a result of increased control of the interferon response. However, in mice the same virus was attenuated in comparison with the virus containing homologous pH1N1 polymerase and NS genes. Levels of viral RNA during single-cycles of replication were lower for the virus with H3N2 NS, and this virus reached lower titres in the lungs of infected mice. Thus, virus with pH1N1 polymerase genes did not increase its virulence by acquiring the H3N2 NS gene segment, and MDCK cells were a poor predictor of the outcome of infection *in vivo*.

## Introduction

Influenza A viruses have a genome composed of eight segments of negative-sense RNA encoding a total of 12 proteins. Influenza A virus genomes can readily mutate both by accumulating point mutations and also by reassortment in which they acquire whole gene segments from another virus in a co-infected host. Many of the functions of influenza virus proteins are carried out in conjunction with other viral or host proteins. For example, the HA and NA genes are functionally linked, and their balance of activities is crucial for virus fitness (Kaverin *et al.*, 2000). In addition, the viral polymerase complex consists of a heterotrimer of three polymerase proteins, PB1, PB2 and PA that interact with each other, the viral nucleoprotein and also viral RNA to form the ribonucleoprotein complex, vRNP (reviewed by Naffakh *et al.*, 2008). Thus, the mixing of influenza virus genes by reassortment may exert unexpected epistatic affects due to the interaction of one gene on another.

The influenza A virus NS1 gene is a major virulence factor encoded by RNA segment 8, which also encodes the nuclear export protein, NEP, sometimes referred to as NS2 (reviewed by Hale *et al.*, 2008). A critical role for NS1 is to counter the induction of the interferon response, which allows the virus a window of opportunity to establish replication in the immunocompetent host. NS1 antagonizes the interferon induction pathway in at least two different ways: firstly, NS1 can mask the virus-associated RNA that is the pathogen-associated molecular pattern (PAMP) detected by cells leading to interferon induction. NS1 is an RNA-binding protein (Wang & Krug, 1996) and it also interferes with the activation of RIG-I, the pattern recognition receptor that detects influenza PAMP (Gack *et al.*, 2009; Mibayashi *et al.*, 2007; Pichlmair *et al.*, 2006). Secondly, NS1 can bind to the host cell mRNA processing factor (CPSF-30) and prevent the nuclear export of newly transcribed mRNAs including the induced beta interferon (IFN-β) message (Noah *et al.*, 2003). The relative importance of each of these mechanisms for any one influenza virus depends on the sequence of NS1, which varies considerably (Hayman *et al.*, 2006; Kochs *et al.*, 2009; Kuo *et al.*, 2010). Sequence variation may affect the cellular localization of NS1 (Melén *et al.*, 2007), which will impact its access to the host molecules; RIG-I is a cytoplasmic detector, whereas CPSF-30 is a nuclear factor. In addition, sequence variation may directly affect the interface with which NS1 binds its cellular partners (Hale *et al.*, 2010b; Steidle *et al.*, 2010; Twu *et al.*, 2007). The identity of the viral polymerase genes also affects the efficacy of the NS1–CPSF-30 interaction. Thus, the H5N1 HK97 NS1 protein bound poorly to CPSF-30 when combined with heterologous polymerase genes of the H3N2 Udorn/72 virus, but this interaction was partially restored in a virus with homologous HK97 polymerase genes. This epistatic effect led to increased IFN control in the wild-type HK97 virus (Twu *et al.*, 2007).

Since its emergence in 2009, the pandemic H1N1 influenza A virus (pH1N1) has been the predominant influenza A virus circulating in humans. However, unlike the pandemic viruses of 1918, 1957 and 1968, the 2009 pandemic strain did not displace all other contemporary circulating human influenza viruses. Indeed, the H3N2 subtype of influenza that has circulated in humans since 1968 continues to be detected in surveillance programmes and this warrants its inclusion in the trimeric seasonal influenza vaccine (WHO, 2011). Given the co-circulation of H3N2 and pH1N1 viruses, reassortment in a co-infected human host is a real possibility. However, due to the epistatic effects described above, the outcome of mixing gene segments derived from these two influenza A virus subtypes is not clear and warrants investigation.

In contrast to NS1 proteins of recently circulating seasonal human viruses, the NS1 of pandemic H1N1 virus appears to lack CPSF-30-binding capacity. Hale *et al.* (2010c) attributed this to three mutations in the C terminus of NS1 and showed that repair of these amino acid differences allowed the recombinant virus better control of IFN induction and other cytokine responses. Interestingly, this did not increase virus pathogenicity. Rather, mice infected with the CPSF-30-positive mutant lost less weight and cleared the virus infection more quickly. Simultaneous mutation at 3 aa is an unlikely evolutionary scenario. However, it is possible that pH1N1 could acquire a CPSF-30-binding competent NS gene by reassortment with another circulating strain of influenza virus. An increase in the efficiency with which the novel virus could control the IFN response might lead to enhanced virulence as has been suggested for the 1918 Spanish influenza (Billharz *et al.*, 2009; Geiss *et al.*, 2002).

In this study, we created an artificial reassortant virus in which the NS gene segment from pH1N1 was replaced with that from a contemporary H3N2 virus, to create a mixture of viral polymerase genes and NS genes such as may occur during a natural reassortment of viruses that co-circulate in humans. We compared the replication capacity of the NS reassortant with that of the parental virus *in vitro* and *in vivo* and found surprisingly conflicting results that suggest the balance between virus replication and sensitivity to the innate immune response may differ in different cell and host systems.

## Results

### The pH1N1 NS1 poorly controls induction of interferon

**Fig. 1.**
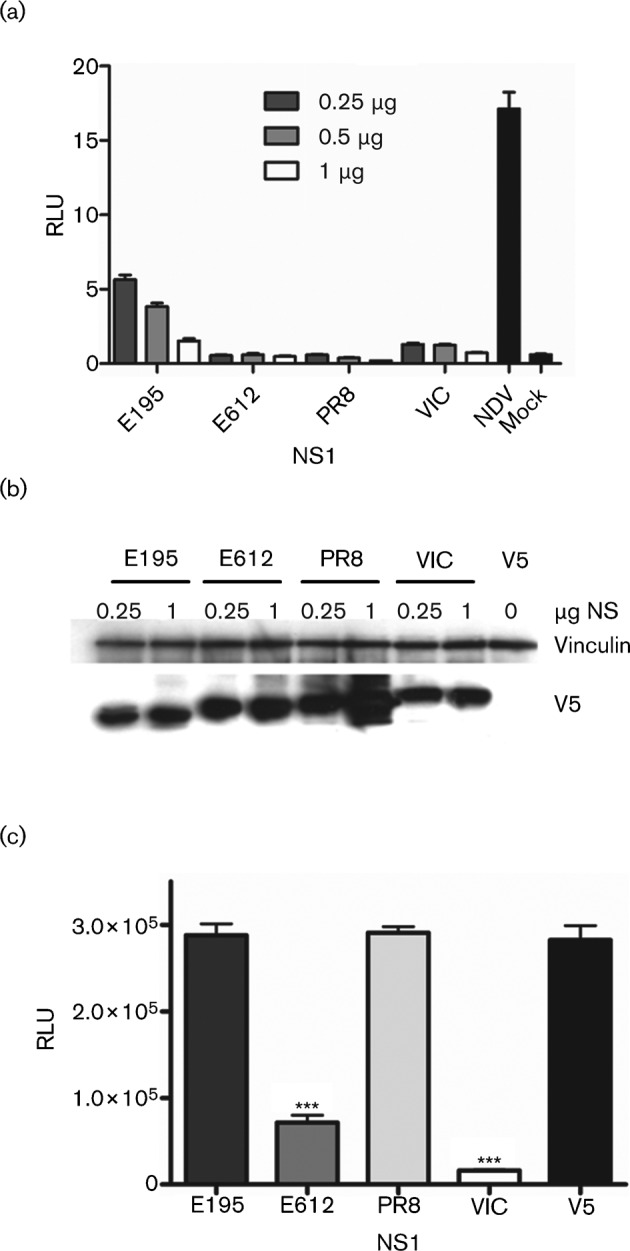
IFN antagonism and cellular localization of NS1. (a) IFN-β induction by NDV was measured in the presence of each NS1 following transfection of three different amounts of plasmid, 0.25, 0.5 and 1 µg DNA as indicated or in the absence of NS1 (V5). Mock was not subjected to NDV infection. Each sample was run in triplicate and the mean is shown with the sd indicated by error bars. This result is representative of at least three different experiments. (b) Samples from the lysates receiving 0.25 or 1 µg DNA in (a) were run on Western blot and probed with anti-V5 tag antibody to determine the level of NS1 protein expressed in the cells. Vinculin was probed as a loading control. (c) NS1 proteins, each with a V5 tag at the C terminus, were expressed in 293T cells and their ability to inhibit host protein expression was determined by their ability to inhibit luciferase expression from a polymerase II promoter. The V5 empty vector indicates luciferase expression in the absence of NS1. The samples were run in triplicate and the mean is plotted with sd indicated by error bars. Statistical analysis using an unpaired Student’s *t*-test was used to determine significance in comparison to the V5 alone luciferase signal (***P≤0.0005). This result is representative of at least three different experiments.

We and others have described differences in the mechanism and efficacy with which NS1 proteins control induction of IFN by influenza virus itself or by exogenous stimuli such as infection with Sendai virus or Newcastle disease virus (NDV) (Hayman *et al.*, 2006; Jiao *et al.*, 2008; Kochs *et al.*, 2007; Kuo *et al.*, 2010; Munir *et al.*, 2011; Park *et al.*, 2003; Wang *et al.*, 2010). The NS1 of pH1N1 virus strain A/California/04/2009 was previously shown to control IFN induction (Hale *et al.*, 2010b, c). We cloned the NS1 protein from a prototype early isolate of pH1N1, A/England/195/09 (E195) whose sequence is identical at the amino acid level to that of A/Cal/04/09. The ability of NS1 from E195 to block expression of luciferase driven from the IFN-β promoter induced by NDV infection in human 293T cells (Hayman *et al.*, 2006) was compared with the inhibition by other NS1 proteins representative of well studied laboratory influenza viruses, A/PR/8/34 (PR8) and A/Victoria/3/75 (VIC) as well as with an NS1 typical of contemporary circulating H3N2 influenza A strains, A/England/612/03 (E612). E195 NS1 was less effective at blocking IFN induction than the other NS1 proteins tested, but this was only evident when low plasmid concentrations were used, Fig. 1(a). Western blot analysis showed that all NS1 proteins were expressed and were of the expected molecular mass, Fig. 1(b).

To attempt to account for the rather weak efficacy of E195 NS1 protein to control IFN induction, we studied its ability to inhibit host gene expression. We monitored expression from a plasmid encoding luciferase downstream of a polymerase II promoter in the presence of NS1, as an indirect measure of NS1’s ability to inhibit host gene expression (Kochs *et al.*, 2007). E195 NS1 did not inhibit luciferase expression, an observation in line with the lack of CPSF-30 binding by this protein (Hale *et al.*, 2010c). In contrast, the H3N2 NS1 protein from the E612 virus behaved like the NS1 protein of VIC and significantly inhibited expression of the reporter gene, Fig. 1(c).

### Recombinant influenza viruses with pH1N1 NS gene do not efficiently control IFN induction

We next generated a panel of recombinant viruses that differed in RNA segment 8 that encodes both NS1 and NEP proteins. One series was based on PR8 virus and contained either PR8 NS, a mutant PR8 NS that lacks RNA-binding capacity in NS1 (PR8 RKS) (Donelan *et al.*, 2003), or the NS from E195 or E612. In the second series, each virus had HA, NA and M gene segments from PR8 to allow equal entry into and egress from infected cells, combined with the polymerase and nucleoprotein genes from pH1N1 E195. In this series, the NS gene segment was derived either from E195, PR8 or E612.

**Fig. 2.**
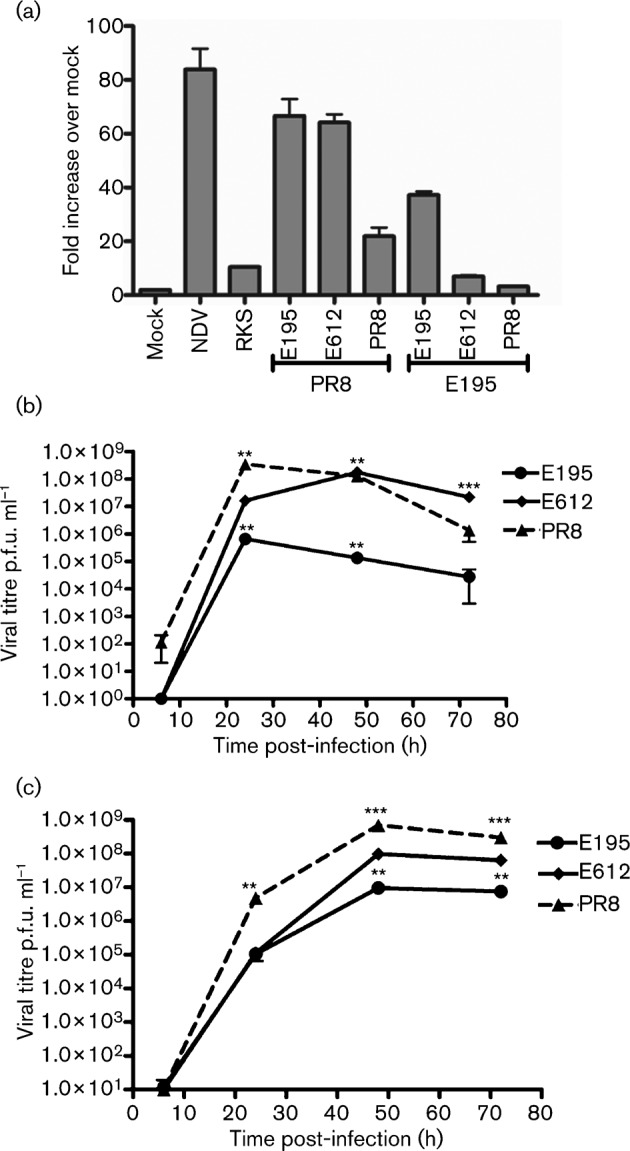
IFN induction and multi-cycle replication of recombinant viruses that differ in NS. All recombinant viruses had the HA, NA and M of A/PR/8/34 and the PB1, PB2, PA and NP genes from either PR8 or A/England/195/09. The NS genes were either from pH1N1 A/England/195/09 (E195), H3N2 A/England/612/03 (E612) or H1N1 A/PR/8/34 (PR8) or PR8 with an RNA-binding mutation (PR8 RKS). (a) Recombinant viruses with PR8 HA, NA and M genes and different polymerase and NS constellations were assessed for induction of IFN-β response by monitoring activation of the IFN-β promoter using the IFN-β luciferase reporter A549 cell line. Relative light units (RLU) indicate luciferase signal produced. The samples were run in triplicate and the mean is plotted with sd indicated by the error bars. This result is representative of at least three different experiments. (b) and (c) A multi-cycle growth curve was performed at 37 °C in (b) MDCK cells or (c) IFN-deficient MDCK-NPro cells, with starting inoculum of an m.o.i. of 0.001. Each virus was assayed in triplicate and graph indicates mean p.f.u. ml^−1^ with error bars correlating to sd. Statistical analyses using an unpaired Student’s *t*-test was used to determine significance in comparison to the E195 growth (**P≤0.005, ***P≤0.0005).

Each virus was used at equal multiplicity to infect an A549 cell line that contains a reporter construct in which the IFN-β promoter is upstream of luciferase, Fig. 2(a) (Hayman *et al.*, 2006). In this system both NDV and the PR8 RKS mutant induced high levels of luciferase in contrast to wild-type PR8. The virus with PR8 polymerase constellation combined with NS from E195 induced a high level of IFN-β, whereas the same polymerase constellation combined with the E612 NS resulted in low levels of IFN-β induction. Similarly, the virus with E195 polymerase and E195 NS gene induced a moderate IFN signal, whereas exchange of the NS gene segment for that of PR8 or E612 significantly reduced the extent to which the IFN-β promoter was induced, Fig. 2(a).

### Recombinant virus with pH1N1 polymerase and H3N2 NS shows increased replication in Madin–Darby canine kidney (MDCK) cells

Next, the viruses with E195 polymerase genes and different NS segments were assayed for multi-cycle replication in MDCK cells. Under these conditions, the switch in NS gene segment had a striking effect on replication, Fig. 2(b). Viruses with either PR8 or E612 NS genes replicated to titres that were 2–3 logs higher at 24, 48 and 72 h post-infection than the virus possessing the pandemic E195 NS segment.

Although MDCK cells are partially IFN incompetent and lack a functioning Mx homologue (Seitz *et al.*, 2010), they do mount an IFN response that limits replication of NS1 deficient or mutated viruses (García-Sastre *et al.*, 1998). We repeated the growth curve of our panel of viruses in a line of MDCK cells in which constitutive expression of the bovine viral diarrhea virus (BVDV) NPro protein targets IRF3 for proteasomal degradation, MDCK NPro (Hilton *et al.*, 2006). In this cell system, that is likely more deficient in its ability to mount an effective IFN response, the difference in replication between viruses with E195 NS and E612 NS was less marked. Although still statistically significant, titres of E195 NS virus were now only 1 log lower than of E612 NS virus. This suggests that the accumulation of antiviral response in normal MDCK cells limited the replication of the E195 NS virus, Fig. 2(c).

### Recombinant virus with pH1N1 polymerase and H3N2 NS generates decreased vRNA during single-cycle replication in A549 cells

**Fig. 3.**
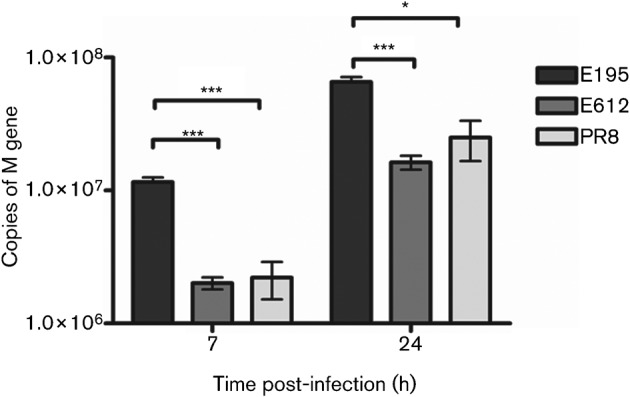
Accumulation of vRNA during single-cycle replication of viruses that differ in NS. Viruses had the HA, NA and M of A/PR/8/34 and the PB1, PB2, PA and NP genes from A/England/195/09. The NS genes were either from pH1N1 A/England/195/09 (E195), H3N2 A/England/612/03 (E612) or H1N1 A/PR/8/34 (PR8). (a) A549cells were infected with an m.o.i. of 2. Seven or 24 h post-infection, cells were lysed and total RNA extracted. The number of vRNA M gene segment copies was determined by qRT-PCR. Each sample was performed in triplicate and the mean is shown with the sd indicated by the error bars. Statistical analyses using an unpaired Student’s *t*-test was used to determine significance when compared to E195 (*P≤0.05, ***P≤0.0005).

We next analysed the accumulation of RNAs produced during single-cycle replication by the three E195 polymerase recombinant viruses that differed in their NS gene segments. M gene segment vRNA was quantified by qRT-PCR, at two time points following infection at high multiplicity in A549 cells, Fig. 3. Acquisition of the NS gene segment from E612 led to reduced amplification of vRNA during replication compared with the virus containing the E195 NS.

### Recombinant virus with pH1N1 polymerase and H3N2 NS is attenuated in mice

**Fig. 4.**
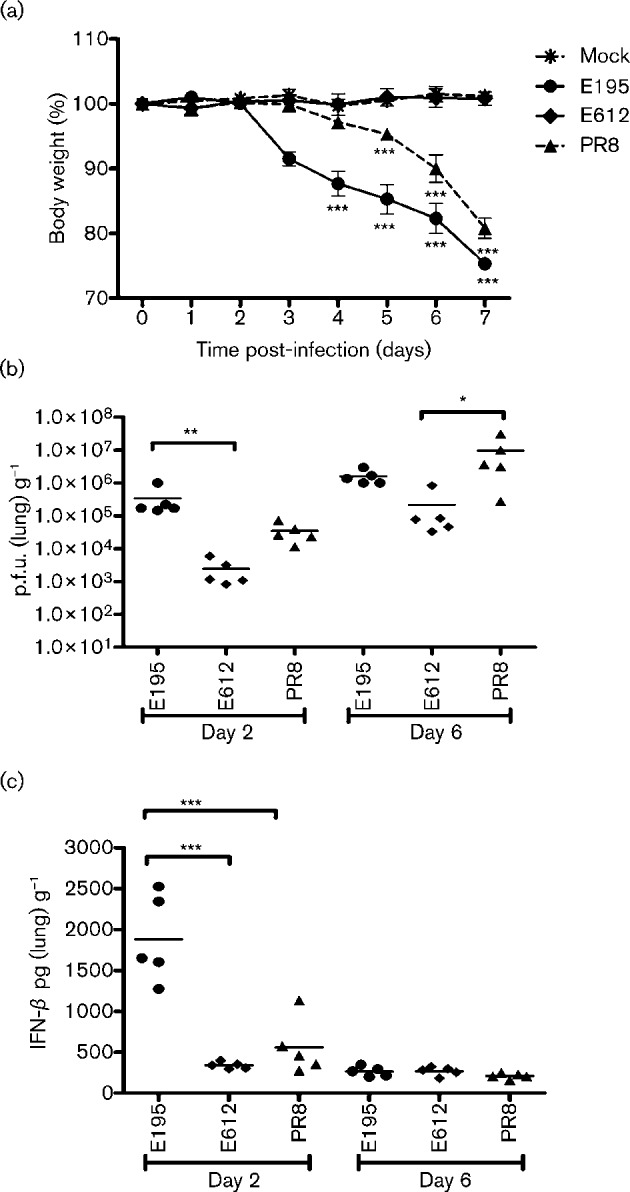
Infection of mice with viruses that differ in NS. Viruses had the HA, NA and M of A/PR/8/34 and the PB1, PB2, PA and NP genes from A/England/195/09. The NS genes were either from pH1N1 A/England/195/09 (E195), H3N2 A/England/612/03 (E612) or H1N1 A/PR/8/34 (PR8). Groups of five mice were inoculated with 1×10^4^ p.f.u. of each virus or PBS (Mock). (a) Mice were monitored daily for weight loss. Mean weight loss is plotted with sE shown by the error bars. Statistical analyses using an unpaired Student’s *t*-test was used to determine significance in comparison to the Mock weight loss (***P≤0.0005). (b) and (c) On days 2 or 6 post-infection one group of mice for each virus were sacrificed. The lungs were harvested and homogenized before titration to determine viral load by plaque assay or amount of IFN-β expression by ELISA. Each animal’s viral load per gram of lung tissue or pg of IFN-β per gram of lung tissue is plotted with mean indicated. Statistical analysis using an unpaired Student’s *t*-test was used to determine significance in comparison to the E195 viral load (***P≤0.0005).

The same three recombinant viruses were then used to infect mice. Virus with homologous pH1N1 polymerase and NS genes induced the highest weight loss. Weight loss occurred later and to a lesser extent for the virus with PR8 NS virus. Infection with the E612 NS virus resulted in no weight loss at all when compared with mice that were mock infected with PBS alone, Fig. 4(a). Titres of infectious virus in the lungs of infected mice were significantly higher at day 2 for the E195 NS virus than for either of the other two viruses, Fig. 4(b). This correlated with a high IFN-β load in the lungs, Fig. 4(c). By day 6 viral titres of the PR8 NS virus had also increased, the likely cause of weight loss in that group from day 5 onwards. Mice infected with recombinant virus with E612 NS did accumulate significant titres of infectious virus (>10^6^ p.f.u. g^−1^) in their lungs by day 6 post-infection, but this was not associated with any measurable IFN response and these mice did not lose weight.

## Discussion

The NS1 gene is a major virulence factor and the NS1 protein of the pH1N1 virus differs from most other naturally occurring NS1 proteins in that, through point mutations and C-terminal truncation, it lacks the ability to bind two host cell factors, CPSF-30 and PABP II, by which NS1 would normally control host gene expression (Hale *et al.*, 2010a; Tu *et al.*, 2011). Thus, we speculated that transfer of the NS gene segment from a circulating H3N2 influenza A virus to a virus with pH1N1 polymerase gene segments might increase virus replication by enhancing its ability to suppress IFN induction. Indeed, in standard MDCK cell culture, used widely in influenza laboratories, this was the case; increased viral titre was observed when the pandemic polymerase constellation was coupled with the H3N2 E612 NS gene, Fig. 2(b). Furthermore, we confirmed that acquisition of the E612 NS gene segment resulted in decreased IFN induction, Fig. 2(a), and speculate that this accounts for the enhanced MDCK replication. However, to our surprise, the outcome of infection with the reassortant viruses in mice was the opposite of what one might have predicted from the *in vitro* growth curves. Infection with the E612 NS virus resulted in no weight loss, probably due to its slow replication *in vivo* and little if any detectable IFN-β induction in the lung. Thus, in this instance MDCK replication was a poor predictor of disease outcome.

Some explanation for these dichotomous results may be found in the quantification of replicated vRNA we performed in A549 cells. The virus with E195 NS accumulated more than fourfold higher levels of vRNA at both 7 and 24 h post-infection than the E612 NS reassortant. The effect of NS segment switching on vRNA output could be due to one or both of the NS segment gene products, NS1 and NEP, affecting polymerase function. Indeed, a role for NEP in regulating transcription and replication of influenza RNAs has been proposed (Robb *et al.*, 2009). There are 14 aa differences between E195 NEP and E612 NEP (data not shown). In addition, it has been known for many years that NS1 regulates the activity of the viral polymerase (Falcón *et al.*, 2004; Marión *et al.*, 1997). More recently an interaction between NS1, CPSF-30 and components of the viral polymerase was suggested (Kuo & Krug, 2009; Twu *et al.*, 2007). Our work indicates that E195 NS1 lacks CPSF-30 binding, whereas E612, a more typical human-adapted NS1, retains the ability to control host gene expression. We suggest that CPSF-30 interaction with NS1 may impose a regulation on polymerase activity that limits RNA accumulation. Thus, viruses that evolve to lose CPSF-30 binding would concomitantly gain an increase in polymerase activity, resulting in higher vRNA yields. This might enable them to continue to replicate even in the face of a moderate cytokine response, a combination which results in enhanced pathogenicity in the mouse model. Indeed in a completely different genetic background, mouse adaptation of the H3N2 influenza strain HK68 resulted in two point mutations that led to loss of CPSF-30 interaction. This virus had an increased replication capacity and also induced high levels of IFN (Dankar *et al.*, 2011). It is also noteworthy that the PR8 virus NS1 protein lacks CPSF-30 binding, a phenotype that was perhaps selected for during mouse adaptation (Hayman *et al.*, 2006; Kochs *et al.*, 2009). It is possible that other viral genes also impact the balance between polymerase activity, NS1 function and IFN antagonism. Notably, it was recently shown that the polymerase genes themselves can control IFN induction (Iwai *et al.*, 2010), a property that may be related to the ability of the PB2 subunit to interact with MAVS in the mitochondria (Graef *et al.*, 2010). In contrast to the outcome of our *in vivo* experiment, Spesock *et al.* (2011) recently showed that acquisition of CPSF-30 binding by the highly pathogenic H5N1 HK/97 virus increased virulence in mice.

In closed systems, under multi-cycle conditions, such as the growth curve we show in Fig. 2(b), accumulation of high levels of IFN will eventually suppress virus replication. However, in mice influenza viruses may induce relatively high levels of IFN and other proinflammatory cytokines that contribute to the demise of the infected animal without sufficiently inhibiting virus replication (Szretter *et al.*, 2007). Whether the same biological consequence of changes in IFN control will play out in the ferret, considered a more authentic model for human influenza, remains to be seen (Meunier & von Messling, 2011) although acquisition of CPSF-30 binding by pH1N1 NS1 mutations attenuated disease in ferrets as well as in mice (Hale *et al.*, 2010c). Indeed, strains of pH1N1 that are more virulent in ferrets also induced a higher level of IFN and proinflammatroy cytokines (Meunier *et al.*, 2012). Importantly, in humans there is a clear correlation between exacerbated cytokine response and severe disease (Peiris *et al.*, 2009).

We showed here that the NS1 of the pH1N1 virus was a comparatively poor antagonist of IFN induction, Fig. 1(a). Since it lacks CPSF-30 binding, the E195 NS1 protein would need to rely on interacting with the viral PAMP and the RIG-I/Trim 25 activation complex to counteract the IFN stimulus. However, pH1N1 NS1 protein is localized to the infected cell nucleus (Tu *et al.*, 2011), suggesting it may not efficiently access RIG-I in the context of virus infection. The sequences that direct nuclear localization of this particular NS1 have not been mapped.

We found that pH1N1 virus induced a moderate amount of IFN in human cells and mice, Fig. 2a and Fig. 4c. Others have suggested this virus induces a weak IFN response (Osterlund *et al.*, 2010). In preliminary studies, we found that several immortalized human cell lines, such as A549 cells, were difficult to infect with pH1N1(unpublished data), which we attribute to an inefficient entry mediated by the HA of this virus, because when we engineered virus with the PR8 HA and NA genes, infection was now efficient (Fig. 2a). We therefore suggest that others have observed low IFN induction signals following pH1N1 infections simply because their target cells were inefficiently infected. In more permissive systems such as human airway cells (Hale *et al.*, 2010c) or mice (Itoh *et al.*, 2009), robust cytokine levels induced by pH1N1 have been detected, supporting the concept that the virus delivers a vigorous PAMP signal which its NS1 is poorly able to control. The mild disease induced in most people infected by pH1N1 virus is incongruous with results obtained in animal models. However, in those who suffered severe outcomes, high viral loads and proinflammatory cytokines were present (To *et al.*, 2010). The work presented here suggests that, as it continues to circulate in its new human host, this virus is unlikely to increase its virulence by acquiring a novel NS gene segment from a current H3N2 virus. Given that NS1 lacking CPSF-30-binding capacity might confer an increase in virulence, it would be important to assess the biological characteristics of a reciprocal reassortant virus in which the H3N2 constellation acquires a novel NS gene from pH1N1.

## Methods

### 

#### Viruses, plasmids and cells.

Viruses were generated via reverse genetics utilizing a 12 plasmid system as described previously (Elleman & Barclay, 2004; Neumann *et al.*, 1999). Viruses E195, PR8, E612 contained the HA, NA and M of A/PR/8/34, the PB1, PB2, PA and NP genes from A/England/195/09 and the NS genes were either from pH1N1 A/England/195/09 (E195), H3N2 A/England/612/03 (E612) or H1N1 A/PR/8/34 (PR8). PR8 WT contained all eight wild-type gene segments and PR8 RKS NS1 had mutations R38A, K41A and S42A.

The NS1 genes from, pH1N1 A/England/195/09 (E195), H3N2 A/England/612/03 (E612), H1N1 A/PR/8/34 (PR8), A/Victoria/3/75 (VIC) or A/HongKong/156/97 (H5N1) were cloned into the pCAGG expression plasmid with a V5 tag encoded at the C terminus of the NS1 protein. The pCAGG plasmid with the V5 tag alone was used as the experimental control (V5).

293T, MDCK, MDCK-NPro, A549, A549-luc and Vero cells were maintained in Dulbecco’s modified Eagle’s medium (DMEM; Gibco, Invitrogen) supplemented with 10 % FBS (Biosera) and 1 % penicillin/streptomycin (Gibco, Invitrogen). A549-luc cells (Hayman *etal.*, 2006) contain a stably transfected luciferase reporter driven by the IFN-β promoter, maintained by supplementation with 2 mg G418 (Gibco, Invitrogen) ml^−1^. MDCK-NPro cells, a kind gift of Professor S. Goodbourn, stably contained an expression plasmid for the BVDV NPro gene, maintained by supplementation of 0.4 mg G418 ml^−1^. All cell lines were grown at 37 °C in 5 % CO_2_.

#### Mouse pathogenicity experiments.

Female BALB/c mice, 6–8-weeks-old, were housed in groups of five and inoculated with 1×10^4^ p.f.u. of virus diluted in PBS or PBS alone (Mock) under isofluorane anaesthetic. Mice were weighed daily and animals were euthanized upon loss of more than 20 % of the start weight. On days 2 and 6 post-infection groups of five animals were sacrificed, the lungs individually harvested and frozen at −80 °C. Lungs were weighed and 500 µl PBS added prior to homogenization. Viral load was assessed by plaque assay of lung homogenates on MDCK cells. The amount of expressed IFN-β in the lung homogenates was assessed by using VeriKine Mouse Interferon Alpha ELISA kit (R&D).

#### Viral growth curve.

Wells of confluent MDCK cells or MDCK-NPro cells were inoculated in triplicate with an m.o.i. of 0.001 for each virus. Following, 1 h of inoculation, at 37 °C, the cells were maintained in serum-free DMEM (Gibco, Invitrogen) containing 1 % penicillin/streptomycin (Sigma-Aldrich) and 1.4 µg TPCK Trypsin (Worthington) ml^−1^. At 24, 48 and 72 h post-infection an aliquot of the media was taken from each well and titrated via plaque assay on MDCK cells. Mean p.f.u. ml^−1^ is plotted with sd as error bars. An unpaired Student's *t*-test was used to assess the statistical significance between viruses.

#### A549 IFN-β luciferase assay.

Triplicate wells of A549-luc cells were inoculated with each virus at an m.o.i. of 3 or with serum-free DMEM for Mock, NDV was used as a positive control. After 1 h of incubation, the inoculum was removed from the cells and replaced with DMEM containing 2 % FBS. Twenty-four hours after inoculation, cells were lysed by using cell culture lysis reagent (CCLR; Promega) and the amount of luciferase quantified using the luciferase assay system (Promega). Mean relative light units (RLU) are plotted with the sd.

#### Inhibition of IFN-β by the NS1 protein.

Triplicate 293T cells in a 12-well-plate were transfected with IFN-β promoter luciferase reporter plasmid, Renilla luciferase expression plasmid and varying amounts (0.25, 0.5 or 1 µg) of NS1 expression plasmids. Twenty-four hours post-transfection, cells were infected with NDV. Twenty-four hours post-infection, cells were lysed using CCLR and luciferase quantified using the dual reporter luciferase assay system (Promega). RLU were normalized to Renilla values and means plotted with the sd of the triplicate as error bars. Equal volumes of lysate were run on 10 % SDS-PAGE and Western blotted for NS1 protein using a mouse anti-V5 antibody (AbD Serotec) and anti-mouse-HRP conjugate (AbD Serotec) or vinculin by using a goat anti-vinculin antibody (Santa Cruz) and anti-goat-HRP conjugate (Santa Cruz).

#### Host gene expression inhibition assay.

Triplicate 293T cells in a six-well-plate were transfected with 0.25 µg luciferase expression plasmid and 1 µg NS1 or the V5 expression plasmid. Twenty-four hours post-transfection, cells were lysed by using CCLR and luciferase quantified using the luciferase assay system (Promega). Mean RLU are plotted and the error bars indicate sd. An unpaired *t*-test was used to assess the statistical significance between viruses.

#### Quantifying viral genome copies.

A549 cells, in triplicate, were infected with an m.o.i. of 1. Following inoculation for an hour at 37 °C virus was removed and 3 % FBS DMEM was added. At either 7 or 24 h post-infection cells were lysed with RLT buffer (Qiagen) and membranes disrupted by spinning through a QIAshredder (Qiagen). Total RNA was purified using an RNeasy mini kit (Qiagen). Equal quantities of total RNA were used to generate influenza vRNA M gene cDNA. An ABI 7900 machine (Applied Biosystems) was then used for qRT-PCR using Taqman primers and probe, specific for the PR8 M gene. A standard curve of PR8 M gene was used to determine genome copy numbers in each sample.
